# Bixin protects mice against ventilation-induced lung injury in an NRF2-dependent manner

**DOI:** 10.1038/srep18760

**Published:** 2016-01-05

**Authors:** Shasha Tao, Montserrat Rojo de la Vega, Hector Quijada, Georg T. Wondrak, Ting Wang, Joe G. N. Garcia, Donna D. Zhang

**Affiliations:** 1 Department of Pharmacology and Toxicology, University of Arizona, Tucson, AZ 85721, USA; 2 Arizona Respiratory Center and Department of Medicine, University of Arizona, Tucson, AZ 85721; 3 Arizona Cancer Center, University of Arizona, 1515 North Campbell Avenue, Tucson, AZ 85724, USA

## Abstract

Mechanical ventilation (MV) is a therapeutic intervention widely used in the clinic to assist patients that have difficulty breathing due to lung edema, trauma, or general anesthesia. However, MV causes ventilator-induced lung injury (VILI), a condition characterized by increased permeability of the alveolar-capillary barrier that results in edema, hemorrhage, and neutrophil infiltration, leading to exacerbated lung inflammation and oxidative stress. This study explored the feasibility of using bixin, a canonical NRF2 inducer identified during the current study, to ameliorate lung damage in a murine VILI model. *In vitro*, bixin was found to activate the NRF2 signaling pathway through blockage of ubiquitylation and degradation of NRF2 in a KEAP1-C151 dependent manner; intraperitoneal (IP) injection of bixin led to pulmonary upregulation of the NRF2 response *in vivo*. Remarkably, IP administration of bixin restored normal lung morphology and attenuated inflammatory response and oxidative DNA damage following MV. This observed beneficial effect of bixin derived from induction of the NRF2 cytoprotective response since it was only observed in *Nrf2*^+/+^ but not in *Nrf2*^−/−^ mice. This is the first study providing proof-of-concept that NRF2 activators can be developed into pharmacological agents for clinical use to prevent patients from lung injury during MV treatment.

Mechanical ventilation (MV) is a life support therapy used to assist patients that have difficulty breathing spontaneously, that present hypoxia or hypotension^1^. It is an intervention procedure for patients suffering from lung trauma, chronic obstructive pulmonary disease (COPD), acute respiratory distress syndrome (ARDS), apnea, severe asthma, or for patients under general anesthesia^2^. Paradoxically, although MV is the only effective strategy to treat these conditions, it may also result in greater lung damage, referred to as ventilator-induced lung injury (VILI), and multi-organ failure that can compromise the patients’ lives^3^,^4^. VILI occurs as an effect of cyclic stretching and overdistension of the lung tissues, which cause severe inflammation and structural tissue damage ultimately leading to acute lung injury (ALI)^5^,^6^. Additional factors that contribute to VILI are the disease or events that led to respiratory failure, and the parameters used in MV treatment (volume, pressure, and duration)^7^. Up to now there are no efficient pharmacological strategies to ameliorate the negative effects caused by MV, and only a conservative approach using a low tidal volume has been shown to cause less damage^8^.

VILI is characterized by a disruption of the alveolar-capillary barrier which increases permeability, thus causing edema, inflammatory leukocyte infiltration (mainly neutrophils), and hemorrhage^9^. Stretch forces cause the release of inflammatory cytokines like IL6, IL8, IL1β, and TNFα by activation of the p38 MAPK pathway and of the transcription factor NF-κB^10^,^11^. Cyclic stretch also generates reactive oxygen species (ROS) that further exacerbate VILI^12^. These events are followed by the onset of an endogenous anti-inflammatory and anti-oxidative reaction to compensate for and attenuate VILI-derived inflammatory response and redox imbalance^13^.

The main cellular cytoprotective response is orchestrated by the transcription factor NRF2 (nuclear factor-E2-related factor 2) which controls the expression of numerous antioxidant, anti-inflammatory, and pro-survival genes that contain antioxidant response elements (ARE) in their promoters^14^,^15^. Typically, NRF2 is ubiquitously expressed and maintained at low levels but is activated quickly in response to various cellular stresses, including mechanical stress^16^,^17^. Importantly, pre-activation of NRF2 facilitates an adaptive response that protects against various types of stresses encountered subsequently. The use of natural compounds to activate NRF2 signaling has proven to be a feasible chemopreventive strategy, as demonstrated in various preclinical studies^18^,^19^,^20^. Many natural compounds that are used in traditional medicine for their antioxidant and anti-inflammatory properties have been shown to be Nrf2 inducers that elicit their effect through NRF2 activation^18^,^21^,^22^. Hence, numerous studies have demonstrated the protective effect of NRF2 against pathologic conditions that present oxidative stress and inflammation^23^,^24^,^25^,^26^. Mechanisms of NRF2 activation by chemopreventive compounds have been studied in detail. Activation of NRF2 in response to chemopreventive compounds or a change in the intracellular redox status is controlled by KEAP1, a substrate adaptor protein of an E3-ubiquitin ligase that binds to NRF2 and negatively regulates it^27^,^28^. Critical cysteine residues in KEAP1 get oxidized by ROS or modified by electrophilic compounds, which then alters its interaction with NRF2 and prevents its degradation^29^,^30^. Interestingly, it has been previously demonstrated by our group that chemopreventive compounds such as sulforaphane (SF) and tert-butylhydroquinone (tBHQ) induce NRF2 in a KEAP1 cysteine-151 (C151)-dependent manner (we defined these inducers as canonical NRF2 inducers). In contrast, carcinogenic arsenic upregulates NRF2 through p62-autophagy blockage (non-canonical mechanism)^29^,^31^.

Bixin is a carotenoid extracted from the seeds of *Bixa orellana* (annatto, or ‘achiote’ in Mexico) used as an FDA-approved food colorant and additive, as well as cosmetic and textile colorant^23^. Traditionally, it has been used in Mexico and South America to treat infectious and inflammatory diseases of the skin, prostate, gastrointestinal tract, and chest pain^32^,^33^. Previous *in vitro* biochemical assays demonstrated that bixin was able to quench singlet oxygen, a ROS implicated in oxidative lung injury^24^,^34^. Consistent with its antioxidant properties, other studies demonstrate that bixin prevents oxidative DNA damage and lipid peroxidation. Bixin also protects against cisplatin-induced clastogenicity and carbon tetrachloride hepatotoxicity^35^,^36^,^37^. Currently, there is no epidemiological evidence of carcinogenicity or acute toxicity associated to ingestion or occupational exposure to bixin, and asides from rare cases of reported allergies to bixin ingestion, this compound has been proven to be safe for human administration^38^,^39^.

In this study, we identified bixin as a novel canonical NRF2 inducer, implying the previously defined antioxidant and anti-inflammatory properties of bixin may be derived from activation of the NRF2-mediated response, rather than acting as a direct ROS scavenger as previously reported. Bixin was found to activate the NRF2 signaling pathway in lung epithelial cells and in the lungs of mice through IP injection. We then explored the protective effects of bixin in a murine VILI model. Bixin protects against VILI by suppression of inflammatory mediators, reduction in alveolar capillary leakage, and protection against DNA oxidative damage in an NRF2-dependant manner. These results suggest that pharmacological activation of NRF2 by bixin pretreatment may ameliorate the lung damage induced by MV, which could constitute the first clinical intervention to prevent VILI.

## Results

### Bixin induces the NRF2 signaling pathway with no detectable toxicity under a wide dose range

**Figure 1:**
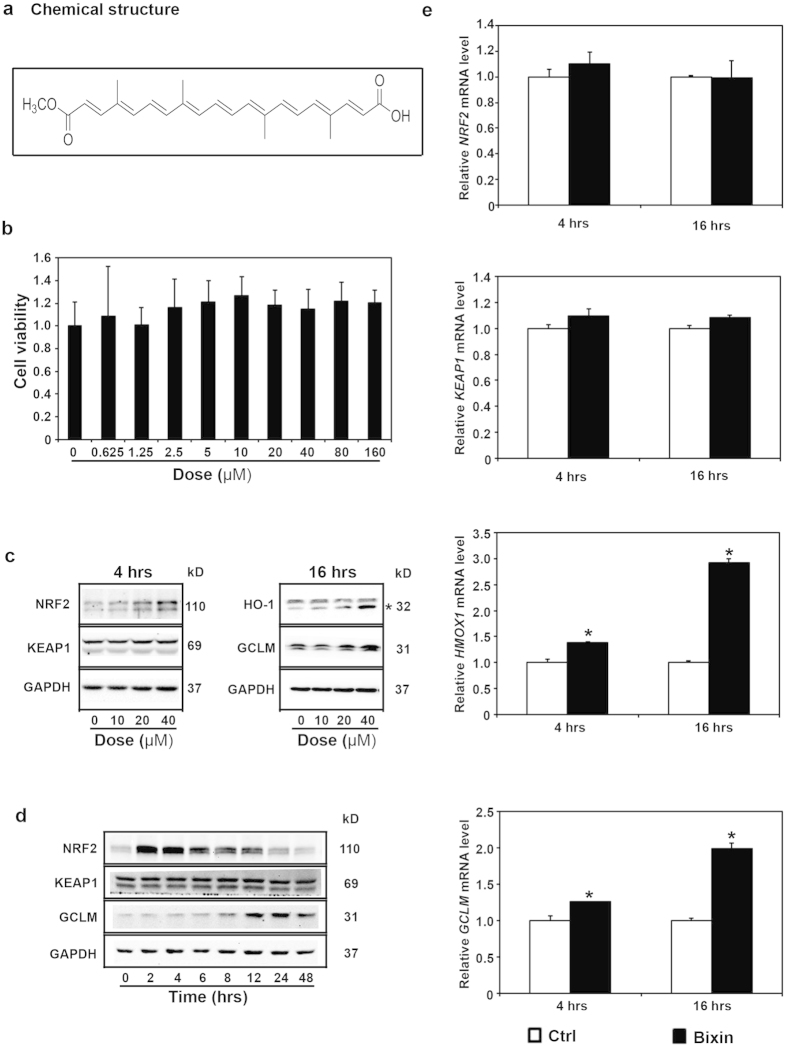
Bixin upregulates the NRF2 signaling pathway. (**a**) Bixin chemical structure. (**b**) Cell viability was measured in H1299 cells treated with the indicated doses of bixin for 48 h. (**c**) H1299 cells were treated with the indicated doses of bixin for 4 h and 16 h, cell lysates were subjected to immunoblot analyses. (**d**) H1299 cells were treated with bixin (40 μM) for the indicated time, cell lysates were subjected to immunoblot analyses. *indicates the specific HO-1 band in H1299 cells. (**e**) H1299 cells were either left untreated (control, Ctrl) or treated with bixin (40 μM) for 4 h and 16 h, and mRNA was extracted. The relative mRNA levels of *NRF2, KEAP1, HMOX1* and *GCLM* were then determined by quantitative real-time RT-PCR. Data are expressed as means ± SD (*p < 0.05, Ctrl *vs.* bixin).

Based on the chemical structure of bixin (Fig. 1a), we investigated if bixin was able to induce the Nrf2 signaling pathway in lung cells. An MTT assay was first employed to determine bixin cytotoxicity in cells treated for 48 h with doses ranging from 0.625–160 μM. In normal primary bronchial epithelial (NHBE) we observed no cytotoxicity at any of the doses tested, whereas in lung microvascular endothelial cells (HMVEC-L) there was a slight decrease in viability (20%) at the highest dose tested (Supplementary Figure S1). In immortalized normal bronchial epithelial cells (HBE and BEAS-2B, data not shown) and the lung cancer cell line H1299 (Fig. 1b) we found no cytoxicity at any of the doses tested. These results demonstrate that bixin is a well-tolerated compound in cells of the lower respiratory system.

Three doses of bixin (10, 20, and 40 μM) were chosen to test their ability to induce the NRF2 signaling pathway in H1299 cells. Immunoblotting analyses show that there was a dose-response effect in the induction of NRF2 protein levels after a 4 h treatment and of its downstream targets HO-1 and GCLM after a 16 h treatment, while no effects were observed on KEAP1 expression levels (Fig. 1c). Since the highest induction was obtained with 40 μM bixin, this dose was then used for a time course study. NRF2 protein levels were significantly induced as early as 2 h after treatment, reaching its highest levels between 2-4 h and returning to basal levels by 24 h (Fig. 1d). This protein induction correlates with increased cytoplasmic and nuclear accumulation of NRF2 (Supplementary Figure S2). In addition, GCLM protein levels increased at 8 h and peaked between 12 and 24 h, with persistent elevation up to 48 h after treatment. As expected, no change was observed in KEAP1 protein levels. Furthermore, 40 μM bixin treatment for either 4 or 16 h did not affect the mRNA levels of *NRF2* nor *KEAP1* (Fig. 1e), consistent with previous observations for canonical NRF2 inducers^31^. In contrast, the mRNA levels of both *HMOX1* and *GCLM* increase significantly after the treatment. These results suggest that bixin is a non-cytotoxic inducer of the NRF2 signaling pathway.

### Bixin is a canonical NRF2 inducer that activates NRF2 in a KEAP1-C151-dependent manner

**Figure 2:**
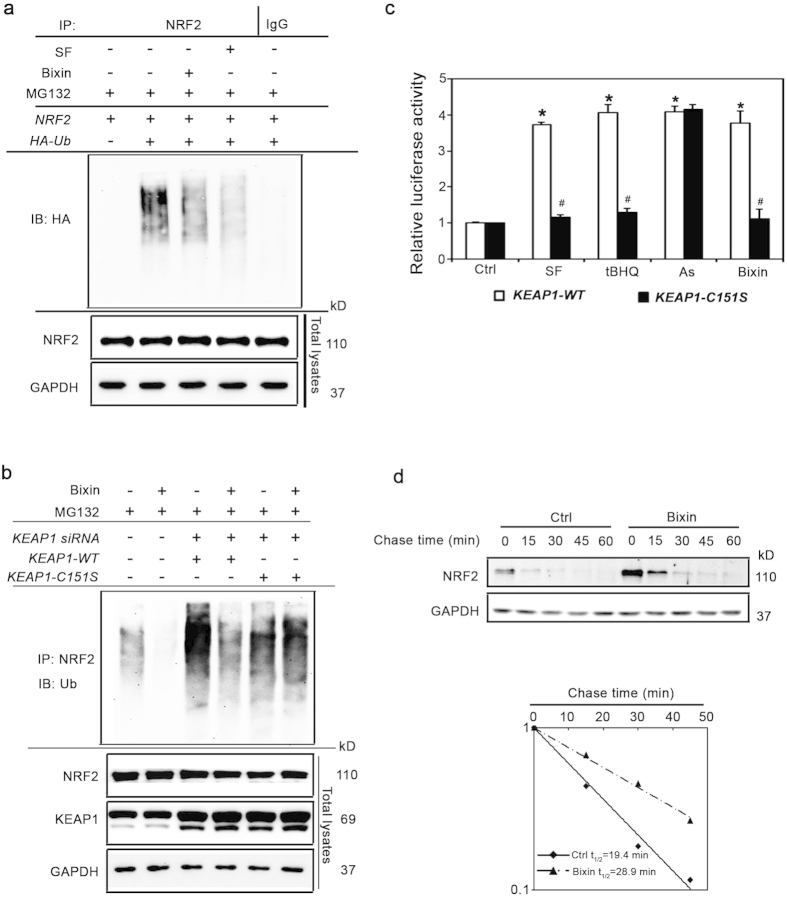
Bixin activates the NRF2 signaling pathway by decreasing NRF2 ubiquitination and increasing NRF2 protein stability in a Keap1-C151-dependent manner. (**a**) H1299 cells were cotransfected with plasmids encoding the indicated proteins; 24 h later the cells were treated with either SF (5 μM) or bixin (40 μM) along with MG132 (10 μM) for 4 h. Anti-NRF2 immunoprecipitates were analyzed by immunoblotting with anti-HA antibody for detection of ubiquitin-conjugated NRF2. (**b**) H1299 cells were transfected with siRNA and 24 h later were transfected with plasmids encoding the indicated KEAP1 proteins. 24 h later the cells were treated with bixin (40 μM) along with MG132 (10 μM) for 4 h. Anti-NRF2 immunoprecipitates were analyzed by immunoblotting with anti-Ub antibody for detection of ubiquitin-conjugated NRF2. (**c**) H1299 cells cotransfected with the plasmids expressing either wild type Keap1 (*KEAP1-WT*) or C151 mutated Keap1 (*KEAP1-C151S*) along with *mGst-ARE* firefly luciferase and *Renilla* luciferase reporters were left untreated or treated with the indicated compounds for 16 h. Dual luciferase activities were measured and the data are expressed as means ± SD (*p < 0.05, Ctrl. *vs.* compound treated groups; ^#^*p* < 0.05, *Keap1-WT vs. Keap1-C151S* group.) (**d**) H1299 cells were either left untreated or treated with bixin (40 μM) for 4 h. Cycloheximide (CHX, 50 μM) was added and cells were lysed at the indicated time points. Cell lysates were subjected to immunoblot analysis using NRF2 and GAPDH antibodies. The intensities of the bands were quantified and plotted against the time after CHX treatment to obtain half-life values.

We next explored the mechanism by which bixin activates the NRF2 pathway. Previous studies have demonstrated that NRF2 inducers cause NRF2 activation by inhibiting its KEAP1-mediated ubiquitination^40^,^41^. Therefore, a cell-based ubiquitination assay was performed in H1299 cells cotransfected with expression vectors for *NRF2* and HA-tagged ubiquitin (*HA-Ub*). The cells were either left untreated or treated with SF (5 μM, as a positive control) or bixin (40 μM), along with the protease inhibitor MG132 (100 μM) for 4 h. Bixin treatment markedly reduced the ubiquitination level of NRF2 compared to the untreated control; as expected, SF treatment also decreased NRF2 ubquitination (Fig. 2a).

Next we tested if bixin is a canonical Nrf2 inducer. H1299 cells were first transfected with *KEAP1*-siRNA to knockdown endogenous protein (Supplementary Figure S3a) and 24 h later were cotransfected with expression vectors for either KEAP1 wild type (*KEAP1-WT*) or a KEAP1 where the cysteine 151 was mutated to serine (*KEAP1-C151S*). The cells were then either left untreated or treated with bixin (40 μM) along with MG132 (100 μM) for 4 h, and the cell lysates were used for a ubiquitination assay. Bixin prevented the ubiquitination of cells with endogenous *KEAP1* or expressing exogenous *KEAP1-WT* but had no effect on cells expressing *KEAP1-C151S* (Fig. 2b). To further confirm that bixin is a canonical NRF2 inducer, endogenous expression of KEAP1 was knocked down in H1299 cells. The cells were then cotransfected with KEAP1-WT or KEAP1-C151S plasmids as well as with *ARE-*firefly luciferase and *Renilla* luciferase reporters to assess NRF2 transcriptional activity. Cells were treated with SF (5 μM), tBHQ (50 μM), As(III) (5 μM), and bixin (40 μM) for 16 h. NRF2 transcriptional activity was enhanced by all treatments in *KEAP1-WT* cells, while in *KEAP1-C151S* cells NRF2 activation by SF, tBHQ, or bixin was inhibited (Fig. 2c). In contrast, arsenic treatment was still able to induce NRF2 transcriptional activity in the *KEAP1-C151S* cells consistent with our previous finding that arsenic is a non-canonical NRF2 inducer that works through a KEAP1 C151-independent mechanism^42^. Taken together, these results demonstrate that bixin is a canonical NRF2 inducer that acts through the critical C151 sensor residue in KEAP1.

Next, the half-life of endogenous NRF2 protein was determined. Cycloheximide was added to untreated or bixin-treated H1299 cells to block *de novo* protein synthesis and cells were harvested at different time points. The protein levels of NRF2 were detected by immunoblot analysis (Fig. 2d, left panel) and the intensity of the NRF2 band was quantified and plotted to calculate the half-life of NRF2 (Fig. 2d, right panel). The half-life of NRF2 of untreated cells was 19.4 min; however, after bixin treatment the half-life of NRF2 increased to 28.9 min. This increase in NRF2 half-life is also KEAP1-C151-dependent (Supplementary Figure S3b). These results indicate that bixin activates NRF2 by decreasing its ubiquitination and increasing NRF2 protein stability in a KEAP1-C151-dependent manner.

### IP injection of bixin activates the NRF2 signaling pathway and suppresses the NF-κB inflammatory response in the lungs of *Nrf2* ^*+/+*^ mice

We first performed a pilot study to test the bixin treatment regimen (dose and injection duration) that resulted in the maximum activation of the NRF2 signaling pathway in the lung. Clearly, IP injection of bixin (200 mg/kg, 72 h) was effective in upregulating pulmonary protein levels of NRF2 and its target genes (*Gclm* and *Hmox*1) in *Nrf2*^+/+^ mice as measured by immunoblot analysis (Supplementary Figure S4). This treatment regimen was then used throughout the study.

**Figure 3:**
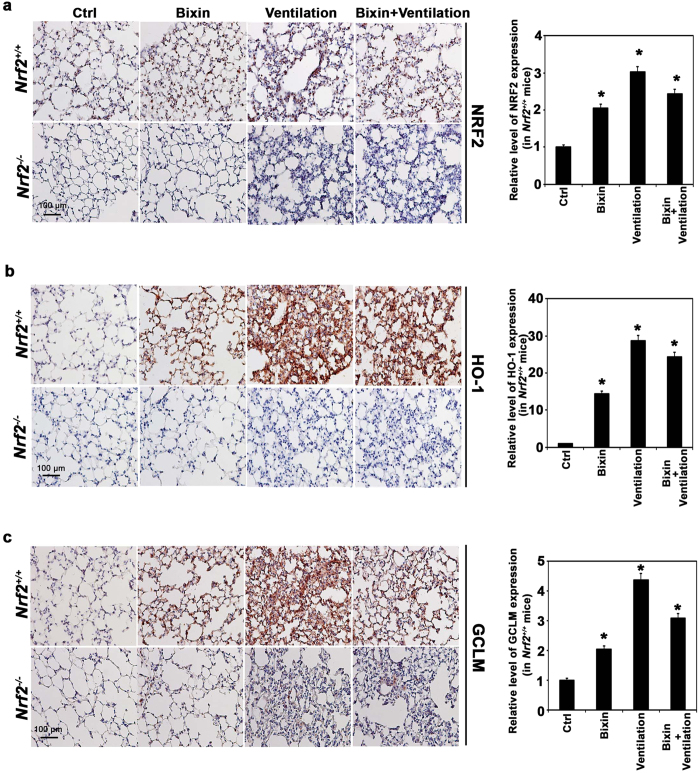
Bixin activates the NRF2 signaling pathway and suppresses the NF-κB inflammatory response in the lungs of *Nrf2*^+/+^ mice. IHC staining of (**a**) NRF2, (**b**) HMOX1, and (c) GCLM of lung tissue sections from *Nrf2*^+/+^ and *Nrf2*^*−/−*^ mice (n = 6, a representative image of the lung tissue from each group is shown to the left). Scale bar: 100 μm. Quantification of relative protein expression (right); results are expressed as means ± SD (*p < 0.05, Ctrl. *vs.* treatment groups).

**Figure 4:**
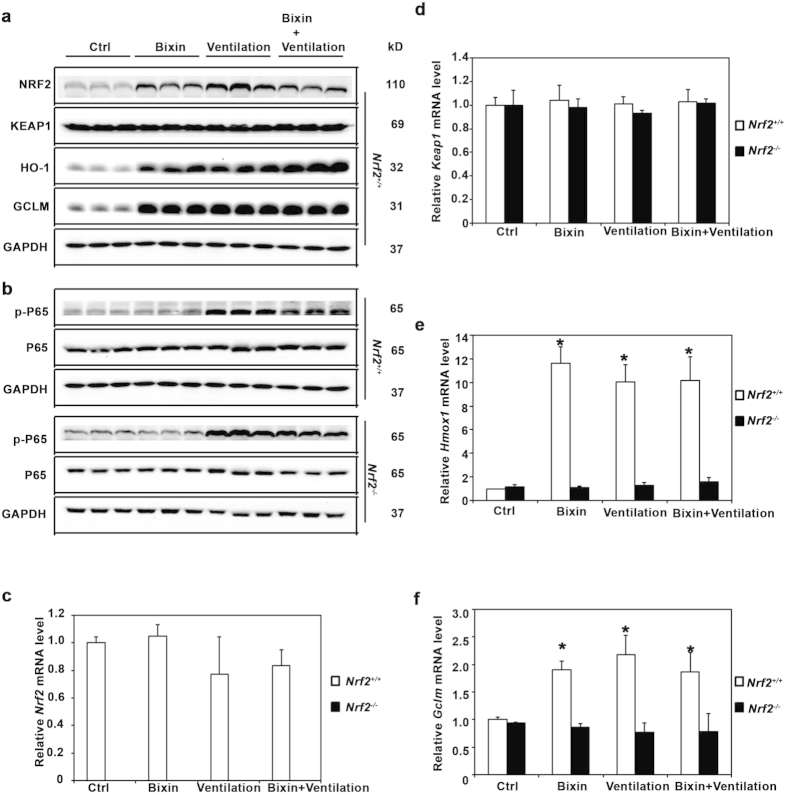
Bixin attenuates ventilation-induced inflammation by inducing the NRF2 signaling pathway and decreasing P65 phosphorylation in the lungs from *Nrf2*^+/^^+^ but not *Nrf2*^*−/−*^ mice. Lung tissue lysates from *Nrf2*^+/+^ mice and *Nrf2*^*−/−*^ mice (n = 3) were subjected to immunoblot analysis with (**a**) NRF2 pathway and (**b**) NF-κB pathway (P65, p-P65) antibodies. The mRNA levels of (**c**) *Nrf2*, (d) *Keap1*, (**e**) *Hmox1,* and (**f**) *Gclm* were measured with RT-PCR assay. Results are expressed as means ± SD (*p < 0.05, Ctrl. *vs.* treatment groups).

The potential protective activity of bixin was studied in a ventilation-induced lung injury (VILI) model. *Nrf2*^+/+^ and *Nrf2*^*−/−*^ mice were IP injected with either corn oil (vehicle control, Ctrl) or bixin (200 mg/kg) 72 h before being subjected to high tidal volume ventilation (40 mL/kg) for 4 h. Lung tissues were collected immediately after ventilation and subjected to immunohistochemistry (IHC) analyses. Indeed, bixin treatment was able to increase NRF2 protein levels (Fig. 3a) as well as HO-1 and GCLM (Fig. 3b,c, respectively) in *Nrf2*^+/+^ mice lungs. As expected, ventilation alone dramatically induced the NRF2 pathway in *Nrf2*^+/+^ mice since mechanical stress is known to induce an NRF2-mediated acute stress response^16^,^17^,^43^ (Fig. 3). Moreover, when the *Nrf2*^+/+^ mice were treated with bixin and ventilation, the NRF2 pathway was also activated (Fig. 3). In contrast, *Nrf2*^*−/−*^mice had no detectable NRF2 and both the basal and inducible levels of HO-1 and GCLM were very low compared to *Nrf2*^+/+^ mice (Fig. 3). Furthermore, immunoblot analyses of total protein extracted from these lung tissues revealed that bixin and ventilation, alone or in combination, can induce NRF2, HO-1 and GCLM in *Nrf2*^+/+^ mice without affecting KEAP1 (Fig. 4a). Since one of the main side effects of ventilation is exacerbated inflammation, the activation of the NF-κB pathway was investigated by detecting phosphorylation of the p65 subunit. While total levels of p65 were unaffected, the phosphorylated (active) form of P65 (p-P65) was markedly induced by ventilation in both *Nrf2*^+/+^ and *Nrf2*^*−/−*^ mice (Fig. 4b). However, bixin pretreatment decreased p-P65 accumulation in *Nrf2*^+/+^ mice, presumably due to the activation of NRF2, with very minimal effects in *Nrf2*^*−/−*^ mice (Fig. 4b). To further corroborate these results, mRNA levels of *Nrf2, Keap1, Hmox1* and *Gclm* were also assessed (Fig. 4c–f). The mRNA levels of *Nrf2* did not increase in the treatment groups, which is consistent with the *in vitro* results demonstrating that bixin activates NRF2 by stabilizing its protein levels (Fig. 4c). As expected, bixin had no effects on the mRNA levels of Keap1 (Fig. 4d). Importantly, although *Hmox1* and *Gclm* had similar basal mRNA levels in *Nrf2*^+/+^ and *Nrf2*^*−/−*^ mice they were only induced after treatment with either bixin, ventilation, or the combination in *Nrf2*^+/+^ mice (Fig. 4e,f).

### Bixin restored normal lung morphology and attenuated inflammatory response and oxidative DNA damage in the lungs of *Nrf2* ^+/+^ but not *Nrf2* ^*−/−*^ mice following MV treatment

**Figure 5:**
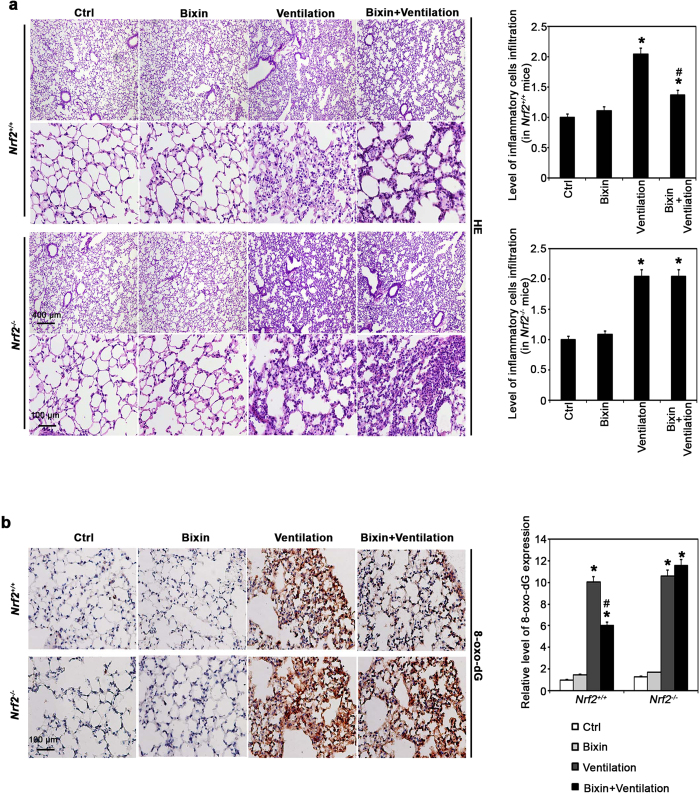
Bixin decreases ventilation-induced inflammation and oxidative DNA damage in the lungs of *Nrf2*^+/^^+^ but not *Nrf2*^*−/−*^ mice. *Nrf2*^+/+^ and *Nrf2*^*−/−*^ mice received IP injection of corn oil or bixin (200 mg/kg, i.p.) 72 h before ventilation treatment for 4 h. (**a**) HE staining and (**b**) IHC of 8-oxo-dG of lung tissue sections from *Nrf2*^+/+^ and *Nrf2*^*−/−*^ mice (n = 6), a representative image (left, amplification:100×, top, and 400×, bottom) of the lung tissues from each group is shown). Scale bar for 100×: 400 μm; 400×: 100 μm. Quantification of inflammatory cells infiltration or relative 8-oxo-dG expression (right); results are expressed as means ± SD (*p < 0.05, Ctrl. *vs.* treatment groups).

Hematoxylin and eosin (HE) staining of lung tissues revealed infiltration of inflammatory cells and alveolar septal thickening in the lungs of both *Nrf2*^+/+^ and *Nrf2*^*−/−*^ mice after 4 h ventilation (Fig. 5a). Bixin injection alone did not affect the lung morphology of mice of either genotype but dramatically attenuated the pulmonary pathological alterations caused by ventilation in *Nrf2*^+/+^ mice, whereas no improvement was observed in the lungs from *Nrf2*^*−/−*^ mice (Fig. 5a). In addition, IHC analysis for 8-hydroxy-2’-deoxyguanosine (8-oxo-dG) was performed to detect ventilation-induced oxidative DNA damage. Ventilation markedly enhanced 8-oxo-dG staining in both *Nrf2*^+/+^ and *Nrf2*^*−/−*^ mice (Fig. 5b). In contrast, bixin treatment alone did not have any effect, indicating it has no pro-oxidant effects at the dose used. However, bixin pretreatment significantly suppressed 8-oxo-dG staining in the lungs from *Nrf2*^+/+^ but not *Nrf2*^*−/−*^ mice that received ventilation (Fig. 5b). These results indicate that bixin protects against ventilation-induced pulmonary damage by decreasing inflammation and oxidative DNA damage, both of which depended on activation of the NRF2 signaling pathway.

**Figure 6:**
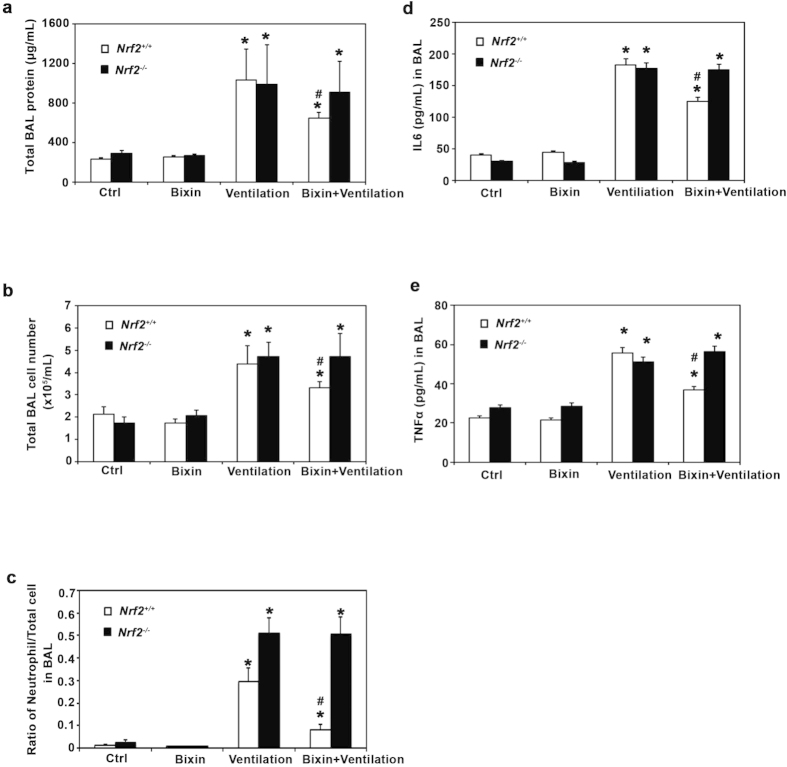
Bixin attenuates ventilation–induced inflammation in the lungs of *Nrf2*^+/^^+^ but not N*rf2*^*−/−*^ mice. (**a**) Total BAL protein, and (**b**) total BAL cell number were measured for *Nrf2*^+/+^ and *Nrf2*^*−/−*^ mice. (**c**) Cell differential analysis was performed on the BAL cells from each mouse. At least 200 cells were counted per sample and the ratio of neutrophils to total cells was plotted. The amount of (**d**) IL6 and (**e**) TNFα in the BAL fluid was measured by ELISA (n = 6). Results are expressed as means ± SD (*p < 0.05, Ctrl. *vs.* ventilation; ^#^*p* < 0.05, ventilation *vs.* bixin + ventilation).

We next analyzed bronchoalveolar lavage (BAL) fluid for total BAL protein, total BAL cell number and ratio of neutrophils. Ventilation greatly increased the total BAL protein in the lungs from both *Nrf2*^+/+^ and *Nrf2*^*−/−*^ mice, which indicated that both genotypes underwent through a similar bronchoalveolar leak (Fig. 6a). Bixin alone did not affect the total protein levels of either mice, but it significantly decreased the total BAL protein in *Nrf2*^+/+^ mice following high tidal volume ventilation (Fig. 6a), which indicates that bixin can suppress ventilation-induced pulmonary vascular leakage in an NRF2-dependent manner. Similarly, ventilation increased inflammatory leukocyte infiltration to the lungs as assessed by the total number of BAL cells and the ratio of neutrophils in both *Nrf2*^+/+^ and *Nrf2*^*−/−*^ mice (Fig. 6). Bixin treatment of unventilated mice did not affect the total cell number or the ratio of neutrophils. However, bixin pretreatment decreased ventilation-induced neutrophil infiltration (total cells and ratio of neutrophils) only in *Nrf2*^+/+^ but not *Nrf2*^*−/−*^ mice (Fig. 6), further suggesting the anti-inflammatory role of NRF2 in bixin-mediated protection against VILI. It is worth mentioning that ventilation-induced neutrophil infiltration was more pronounced in *Nrf2*^*−/−*^ mice (Fig. 6c), indicating the basal level of Nrf2 was sufficient to confer protection against MV-induced inflammatory cell infiltration. Moreover, the amount of inflammatory cytokines (IL6, TNFα) was measured by ELISA as surrogate markers for NF-κB signaling activation. Ventilation greatly induced the expression of IL6 and TNFα in both *Nrf2*^+/+^ and *Nrf2*^*−/−*^ mice while bixin pretreatment reduced the expression of both cytokines only in ventilated *Nrf2*^+/+^ mice (Fig. 6d,e). Collectively, these results suggest that bixin can decrease the pulmonary inflammatory response associated with ventilation through activation of the NRF2 pathway and attenuation of NF-κB signaling.

## Discussion

VILI is a negative side effect of MV that contributes to patient morbidity and mortality despite being the most effective therapy against respiratory deficiencies. In the pathology of VILI, two major events have been identified: volutrauma (damage induced by high respiratory volumes) and biotrauma (damage induced by the mechanical stretching of the airways that produces an inflammatory response)^44^. The first event can be partially reversed by using low tidal volumes for ventilation. However, there are currently no treatments to decrease the biotrauma, but we believe that targeting the biological events and signaling pathways involved in it could constitute a therapeutic option. In this study, the feasibility of using NRF2 activators in decreasing VILI using the newly identified canonical NRF2 activator bixin was clearly demonstrated. First, bixin was identified as an NRF2 pathway activator *in vitro* (Fig. 1). Bixin works in a KEAP1-C151-dependent fashion to prevent NRF2 ubiquitination and prolong its half-life, and is therefore defined as a canonical NRF2 activator (Fig. 2). Thus, it is plausible that the previously reported antioxidant activity of bixin is actually through NRF2 activation. Next, the *in vivo* protective effects of bixin in ameliorating VILI were investigated. As expected, mechanical ventilation itself induced NRF2, HO-1 and GCLM, which is in agreement with the role of NRF2 signaling as a stress response. However, it also elicited a severe inflammatory response (as measured by p-P65 protein levels, total BAL protein, increased BAL cells and neutrophils, and increased levels of inflammatory cytokines) and oxidative stress (as measured by DNA oxidative damage) (Figs 3, 4, 5, 6). Bixin induces the expression of NRF2 and its downstream targets in lung tissues of *Nrf2*^+/+^ mice (Fig. 3). More importantly, bixin pretreatment restored normal lung morphology and alleviated MV-induced inflammation and oxidative stress, these effects seem to be dependent on NRF2 signaling since *Nrf2*^*−/−*^ mice did not benefit from bixin pretreatment (Figs 3, 4, 5, 6).

Although NRF2 is recognized as a major antioxidant and anti-inflammatory factor and its beneficial effects in lung diseases have been previously reported^20^,^45^,^46^ few studies have investigated the role of NRF2 in VILI. Our group and others have identified that VILI upregulates the NRF2 response in lung tissues^16^,^17^ and that genetic ablation of *Nrf2* increases inflammation and oxidative injuries in mice. Papaiahgari *et al.* found that VILI produced high alveolo-endothelial permeability (total BAL protein) in both *Nrf2*^+/+^ and *Nrf2*^*−/−*^ mice to a similar degree^16^. Interestingly, they also reported that *Nrf2*^*−/−*^ mice had a higher neutrophil infiltration to the lungs than their wild-type counterparts. Both observations are consistent with this study. We believe this neutrophil accumulation could account for the increased oxidative damage observed in the lung tissues of *Nrf2*^*−/−*^ mice that received ventilation. Another study found that sodium sulfide protects against VILI by upregulating NQO1 and GPX2^47^, which are involved in the restoration of redox balance. Additionally, hyperoxia causes acute lung injury (ALI), which resembles VILI in that it causes lung hyperpermeability and inflammation by induction of NF-κB and pro-inflammatory cytokine release^11^. Another study has identified that conditional deletion of NRF2 in lung epithelial cells causes greater lung injury and prolonged inflammation, as well as increased alveolar permeability, under hyperoxic conditions^45^. These studies support the hypothesis that hyperoxia is an effect of MV and that NRF2 activation protects the lungs from VILI and ALI by inducing the transcription of antioxidant proteins and by downregulating NF-κB signaling^46^,^48^, consistent with our results.

These studies set the basis for considering NRF2 as an attractive, druggable target to prevent VILI, ALI, and other airway diseases. Since MV is a procedure that can be anticipated, it may be possible to start preventive therapy that induces NRF2 before the procedure is initiated to decrease its negative side effects^4^. Although the use of direct antioxidants, like N-acetyl cysteine (NAC), has some degree of beneficial effects^16^ we suggest that activating the body’s own defensive responses through upregulation of the NRF2 pathway in combination with low tidal ventilator strategies will result in greater benefits for the patients. The use of carotenoids in chemopreventive interventions has been extensively documented although the results have proven little to no effect, probably due to their limited action as ROS quenchers^49^,^50^,^51^,^52^,^53^. Our results suggest that bixin administration before MV might improve the patients’ outcomes thanks to not only its quenching properties but also to its ability to upregulate the antioxidant and anti-inflammatory responses. In humans, the maximum bixin concentration in blood plasma is detected at 2 h post-ingestion^54^, so bixin administration could be a good option even for patients receiving emergency MV. A major concern in the administration of carotenoids is that at high doses they have pro-oxidant effects, however the bixin doses used in our *in vivo* studies did not elicit any cytotoxicity or generate oxidative DNA damage while inducing a robust NRF2 response. All the evidence presented in this study demonstrates that bixin alleviates VILI by induction of NRF2 to decrease inflammation and oxidative damage. Therefore, this study suggests that pharmacological activation of NRF2 with natural compounds such as bixin may prove beneficial to patients who will receive MV treatment. It is also possible that the beneficial effects identified in the lung might also be present in other vital organs (like kidneys) affected by the negative side effects of MV, since IP injection of NRF2 inducers is able to activate NRF2 in many organs tested (data not shown). Certainly, further investigations of the efficacy of bixin in protecting human lung injury are needed. However, this study provides proof-of-concept that NRF2 activators can be developed into pharmacological agents for clinical prevention of lung injury for patients undergoing MV treatment.

## Materials and Methods

### Chemicals, antibodies, and cell culture

Bixin, tBHQ, and sodium arsenite (As(III)) were purchased from Sigma, and sulforaphane (SF) was from Santa Cruz. Primary antibodies against NRF2, KEAP1, GCLM, HO-1, GAPDH, and the hemagglutinin (HA) epitope, as well as horseradish peroxidase (HRP)-conjugated secondary antibodies were from Santa Cruz. Antibodies against p-P65 and P65 were from Cell Signaling, and the 8-oxo-dG antibody was from Trevigen. The Alexa Fluor 488-conjugated secondary antibody was from Invitrogen. The human non-small cell carcinoma H1299 cells were purchased from ATCC and were grown in RPMI 1640 medium supplemented with 10% FBS (Atlanta Biological) and 0.1% gentamycin (Invitrogen). Normal human bronchial epithelial cells (NHBE) and normal lung microvascular endothelial cells (HMVEC-L) were purchased from Lonza and were grown in Bronchial Epithelial Growth Medium (BEGM, Lonza) and Endothelial Cell Growth Medium (EGM, Lonza), respectively, according to the supplier’s instructions. All cells were maintained at 37 °C in a humidified incubator containing 5% CO_2_.

### Transfection of siRNA, cDNA, and luciferase reporter gene assay

Transfection of small interfering RNA (Control siRNA #1027281, *KEAP1* siRNA #SI03246439, Qiagen) was performed using HiPerfect (Qiagen) according to the manufacturer’s instructions. Transfection of cDNA was performed 24 h after siRNA transfection using Lipofectamine 3000 (Invitrogen) according to the manufacturer’s instructions. Activation of NRF2 transcriptional activity was performed as previously published^31^. Briefly, H1299 cells were cotransfected with expression vectors for either *KEAP1* wild type (*KEAP1-WT*) or a mutant *KEAP1* (*KEAP1-C151S*), along with *mGst-ARE* firefly and *Renilla* luciferase reporters. At 24 h post-transfection, cells were treated with SF (5 μM), tBHQ (50 μM), As (5 μM), or bixin (40 μM) for 16 h, then lysed for analysis of the reporter gene activity using the Promega dual-luciferase reporter gene assay system.

### Cell viability

Bixin toxicity was measured by functional impairment of the mitochondria using 3-(4,5-dimethylthiazol-2-yl)-2,5-diphenyltetrazolium bromide (MTT) (Sigma). Cells (1 × 10^4^) were seeded in a 96-well plate; 24 h later the cells were treated with the indicated doses of bixin for 48 h. 20 μL of 2 mg/mL MTT were directly added to the cells which were then incubated at 37 °C for 2h. 100 μl of isopropanol/HCl were added to each well and the plate was shaken at room temperature (RT). Absorbance was measured at 570 nm using the Synergy 2 Multi-Mode Microplate Reader (Biotek).

### Immunoblot analysis, ubiquitylation assay, and protein half life

H1299 cells were harvested in sample buffer (50 mM Tris-HCl [pH 6.8], 2% SDS, 10% glycerol, 100 mM DTT, and 0.1% bromophenol blue), boiled and sonicated. Total cell lysates were resolved by SDS-PAGE and subjected to immunoblot analyses with the indicated antibodies. For the ubiquitination assay, cells were cotransfected with expression vectors for *NRF2 *and HA-tagged ubiquitin (*HA-Ub*), or with *KEAP1* siRNA plus *KEAP1-WT* or *KEAP1-C151S*. The cells were left untreated or treated with either SF (5 μM) or bixin (40 μM) along with MG132 (10 μM) for 4 h. The cells were harvested in buffer containing 2% SDS,150 mM NaCl, 10 mM Tris-HCl (pH 8.0), and 1 mM DTT and boiled. For immunoprecipitation, 1 μg of NRF2 antibody was incubated with the cell lysates at 4 °C overnight with protein A agarose beads (Invitrogen). Immunoprecipitated complexes were washed four times with RIPA buffer and eluted in sample buffer by boiling for 5 min. Samples were resolved by SDS-PAGE and immunoblotted with HA or Ub antibodies. To measure the half-life of NRF2, H1299 cells were either left untreated or treated with bixin (5 μM) for 4 h, then cycloheximide (50 μM) was added to block protein synthesis. Total cell lysates were collected at different time points and subjected to immunoblot analysis with NRF2 antibody. The relative intensity of the bands was quantified using the ChemiDoc CRS gel documentation system and Quantity One software (BioRad).

### mRNA extraction and real-time RT-PCR

Total RNA was extracted from H1299 cells and mouse lung tissues using TRIzol (Invitrogen). Equal amounts of mRNA were used to generate cDNA using the M-MLV Reverse Transcriptase synthesis kit according to the manufacturer’s instructions (Promega). RT-PCR and primer sequences of *NRF2, KEAP1, GCLM, HMOX1* and *GAPDH* were described previously^40^ to evaluate mRNA expression using the LightCycler 480 system (Roche). Quantification of cDNA amount for mouse *Nrf2, Keap1, Gclm*, and *Hmox1* in each lung tissue sample was performed with KAPA SYBR FAST qPCR Kit (Kapa Biosystems). Primers were designed with Primer 3 (http://www-genome.wi.mit.edu/genome_software/other/primer3.html) and synthesized by Sigma as follows:

*Nrf2*: forward (CTCAGCATGATGGACTTGGA)

reverse (TCTTGCCTCCAAAGGATGTC);

*Keap1*: forward (GATCGGCTGCACTGAACTG)

reverse (GGCAGTGTGACAGGTTGAAG);

*Hmox1*: forward (GAGCCTGAATCGAGCAGAAC)

reverse (CTCGGCTTGGATGTGTACCT);

*Gclm*: forward (TCCCATGCAGTGGAGAAGAT)

reverse (AGCTGTGCAACTCCAAGGAC);

*β-actin*: forward (AAGGCCAACCGTGAAAAGAT)

reverse (GTGGTACGACCAGAGGCATAC).

The real-time PCR conditions used were: initial denaturation (95 °C, 3 min), 40 cycles of amplification (95 °C, 10 s; 60 °C, 20 s; 72 °C, 5 s), melting curve (95 °C, 5 s; 65 °C, 1 min; 97 °C continuous), and cooling cycle (40 °C, 30 s). Mean crossing point (Cp) values and standard deviations (SD) were determined. Cp values were normalized to the respective Cp values of the mouse *β-actin* reference gene. Data are presented as a fold change in gene expression compared to the control group.

### Animals and treatments

*Nrf2*^+/+^ and *Nrf2*^*−/−*^ SKH-I mice were obtained by breeding *Nrf2*^+*/−*^ mice. All animals received water and food ad libitum, were handled according to the Guide for the Care and Use of Laboratory Animals, and the protocols were approved by the University of Arizona Institutional Animal Care and Use Committee. Eight-week-old *Nrf2*^+/+^ and *Nrf2*^*−/−*^ mice were randomly allocated into four groups (n = 6): (i) control (corn oil); (ii) bixin (200 mg/kg, dissolved in corn oil); (iii) ventilation; (iv) bixin+ventilation. Bixin was administrated through intraperitoneal (IP) injection 72 h before ventilation. For ventilation-induced lung injury (VILI) experiments, mice were subjected to mechanical ventilation^9^. Briefly, mice were anesthetized with ketamine/xylazine (IP, 100/5 mg/kg, respectively), intubated with a 20-gauge IV catheter, and connected to the ventilator (Inspira, Harvard Apparatus). The ventilation parameters using room air were: tidal volume 40 mL/kg, respiratory rate 75 breaths/min, and a positive and expiratory pressure of 0 cm H_2_O for 4 h. Mice were constantly monitored and deep anesthesia was maintained throughout the experiment with ketamine/xylazine. Mice in the control and bixin groups were allowed to breathe spontaneously. All mice survived the ventilation treatment and/or bixin injections.

### Bronchoalveolar lavage (BAL) and lung tissue collection

After the treatments, mice were euthanized and BAL fluid was obtained by lavaging the lung with 1 mL HBSS (Invitrogen) through the tracheal cannula^9^. The BAL fluid was centrifuged at 500× g for 20 min at 4 °C to collect the cells. Cell pellets were resuspended in PBS and total cell counts were determined using the TC20 automated cell counter (BioRad). Cytospins of BAL cells were prepared (Cytospin 4, Thermo Fisher Scientific) and slides were stained with the Shandon Kwik-Diff kit (Thermo Fisher Scientific). Macrophages and neutrophils were identified using the standard morphologic criteria; 200 cells were examined per sample. The mean cell counts ± SD were obtained from 6 mice of each group. The supernatant collected from the BAL fluid was centrifuged again at 15,000 x g for 10 min at 4 °C and stored at –80 °C until used for protein analysis. Lungs were collected and divided: one part was frozen in liquid nitrogen for total RNA extraction and protein analysis; the other part was fixed in 10% buffered formalin and embedded in paraffin for histological and immunochemical analyses.

### HE staining and IHC

Tissue sections (4 μm) were baked and deparaffinized. Hematoxylin and eosin (HE) staining was performed for pathological examination. IHC analysis was performed as previously described^40^. Briefly, antigen retrieval was performed by boiling the slides with retrieval solution (citric acid monohydrate 2.1 g/L in H_2_O, pH = 6.0) three times for 5 min. Tissue sections were then exposed to 3.5 M HCl for 15 min at room temperature and washed with PBS. Subsequently, tissue sections were treated with 0.3% peroxidase to quench endogenous peroxidase activity. Tissue sections were incubated with 5% normal goat serum for 30 min followed by 2 h incubation with NRF2 antibody at 1:100 dilution at RT. Staining was performed using the EnVision + System-HRP kit (Dako) according to the manufacturer’s instructions.

### Enzyme-linked immunosorbent assay (ELISA) of cytokines in BAL fluid

The ELISA kit (eBiosciences) was used according to the manufacturer’s instructions. Briefly, the plate was coated with 100 μL capture antibody in coating buffer per well and incubated overnight. The plate was washed with 250 μL wash buffer, blocked with 200 μL of the assay diluents, and incubated for 1 h. BAL fluid (100 μL) was added and incubated for 2 h, then 100 μL of detection antibody (IL-6, TNFα) were added to each well and incubated for 1 h. Subsequently, 100 μL avidin-HRP were added and the plate was incubated for 30 min. 100 μL of the substrate solution were added to each well and incubated for 15 min; the reaction was stopped with 50 μL of stop solution. All incubations were done at RT. The plate was read at 450nm.

### Indirect immunofluorescence

H1299 cells were seeded on glass cover slips, 24h later they were treated with bixin (40 μM) for the indicated time points. Cells were fixed with chilled methanol and incubated with anti-NRF2 antibody, then with an Alexa Fluor 488-conjugated secondary antibody. Nuclei were counterstained with DAPI. Images were obtained using a Zeiss Observer.Z1 microscope with the Slidebook 5.0 software (Intelligent Imaging Innovations).

### Statistics

Results are presented as the mean ± SD of three independent experiments performed in duplicate (real-time RT-PCR) or triplicate. Statistical tests were performed using SPSS 13.0. Unpaired Student’s t-tests were used to compare the means of two groups. One-way ANOVA with Bonferroni’s correction was used to compare the means of three or more groups. *P* < 0.05 was considered to be significant.

## Additional Information

**How to cite this article**: Tao, S. *et al.* Bixin protects mice against ventilation-induced lung injury in an NRF2-dependent manner. *Sci. Rep.* **6**, 18760; doi: 10.1038/srep18760 (2016).

## Supplementary Material

Supplementary Information

## References

[b1] Rojo A. I. *et al.* The PTEN/NRF2 axis promotes human carcinogenesis. Antioxid Redox Signal 21, 2498–514 (2014). 24892215 10.1089/ars.2014.5843 PMC4245871

[b2] Son Y. O. *et al.* Nrf2/p62 signaling in apoptosis resistance and its role in cadmium-induced carcinogenesis. J Biol Chem 289, 28660–75 (2014). 25157103 10.1074/jbc.M114.595496 PMC4192515

[b3] Strohecker A. M. & White E. Autophagy promotes BrafV600E-driven lung tumorigenesis by preserving mitochondrial metabolism. Autophagy 10, 384–5 (2014). 24362353 10.4161/auto.27320 PMC5396093

[b4] Luis A. *et al.* Oxidative stress-dependent activation of the eIF2alpha-ATFr unfolded protein response branch by skin sensitizer 1-fluoro-2,4-dinitrobenzene modulates dendritic-like cell maturation and inflammatory status in a biphasic manner. Free Radic Biol Med 77, 217–29 (2014). 25236743 10.1016/j.freeradbiomed.2014.09.008

[b5] Tanida I. Autophagosome formation and molecular mechanism of autophagy. Antioxid Redox Signal 14, 2201–14 (2011). 20712405 10.1089/ars.2010.3482

[b6] Klionsky D. J. *et al.* Guidelines for the use and interpretation of assays for monitoring autophagy. Autophagy 8, 445–544 (2012). 22966490 10.4161/auto.19496 PMC3404883

[b7] Levine B. & Kroemer G. Autophagy in the pathogenesis of disease. Cell 132, 27–42 (2008). 18191218 10.1016/j.cell.2007.12.018 PMC2696814

[b8] Wakabayashi N. *et al.* Notch-Nrf2 axis: regulation of Nrf2 gene expression and cytoprotection by notch signaling. Mol Cell Biol 34, 653–63 (2014). 24298019 10.1128/MCB.01408-13 PMC3911489

[b9] Li L. *et al.* SQSTM1 is a pathogenic target of 5q copy number gains in kidney cancer. Cancer Cell 24, 738–50 (2013). 24332042 10.1016/j.ccr.2013.10.025 PMC3910168

[b10] Wang W. *et al.* Nrf2 enhances myocardial clearance of toxic ubiquitinated proteins. J Mol Cell Cardiol 72, 305–15 (2014). 24747945 10.1016/j.yjmcc.2014.04.006 PMC4418517

[b11] Ray P. D., Yosim A. & Fry R. C. Incorporating epigenetic data into the risk assessment process for the toxic metals arsenic, cadmium, chromium, lead, and mercury: strategies and challenges. Front Genet 5, 201 (2014). 25076963 10.3389/fgene.2014.00201 PMC4100550

[b12] White E. The role for autophagy in cancer. J Clin Invest 125, 42–6 (2015). 25654549 10.1172/JCI73941 PMC4382247

[b13] Zhang C. F. *et al.* Suppression of autophagy dysregulates the antioxidant response and causes premature senescence of melanocytes. J Invest Dermatol 135, 1348–57 (2015). 25290687 10.1038/jid.2014.439

[b14] Jaramillo M. C. & Zhang D. D. The emerging role of the Nrf2-Keap1 signaling pathway in cancer. Genes Dev 27, 2179–91 (2013). 24142871 10.1101/gad.225680.113 PMC3814639

[b15] Kensler T. W., Wakabayashi N. & Biswal S. Cell survival responses to environmental stresses via the Keap1-Nrf2-ARE pathway. Annu Rev Pharmacol Toxicol 47, 89–116 (2007). 16968214 10.1146/annurev.pharmtox.46.120604.141046

[b16] Papaiahgari S. *et al.* Genetic and pharmacologic evidence links oxidative stress to ventilator-induced lung injury in mice. Am J Respir Crit Care Med 176, 1222–35 (2007). 17901416 10.1164/rccm.200701-060OC PMC2176106

[b17] Mirzapoiazova T. *et al.* Non-muscle myosin light chain kinase isoform is a viable molecular target in acute inflammatory lung injury. Am J Respir Cell Mol Biol 44, 40–52 (2011). 20139351 10.1165/rcmb.2009-0197OC PMC3028257

[b18] Shen T. *et al.* Plant extracts of the family Lauraceae: a potential resource for chemopreventive agents that activate the nuclear factor-erythroid 2-related factor 2/antioxidant response element pathway. Planta Med 80, 426–34 (2014). 24585092 10.1055/s-0034-1368197 PMC4393250

[b19] Tao S., Justiniano R., Zhang D. D. & Wondrak G. T. The Nrf2-inducers tanshinone I and dihydrotanshinone protect human skin cells and reconstructed human skin against solar simulated UV. Redox Biol 1, 532–41 (2013). 24273736 10.1016/j.redox.2013.10.004 PMC3836278

[b20] Zheng Y. *et al.* Sulforaphane prevents pulmonary damage in response to inhaled arsenic by activating the Nrf2-defense response. Toxicol Appl Pharmacol 265, 292–9 (2012). 22975029 10.1016/j.taap.2012.08.028 PMC3725323

[b21] Surh Y. J. Cancer chemoprevention with dietary phytochemicals. Nat Rev Cancer 3, 768–80 (2003). 14570043 10.1038/nrc1189

[b22] Lau A., Whitman S. A., Jaramillo M. C. & Zhang D. D. Arsenic-mediated activation of the Nrf2-Keap1 antioxidant pathway. J Biochem Mol Toxicol 27, 99–105 (2013). 23188707 10.1002/jbt.21463 PMC3725327

[b23] Cullup T. *et al.* Recessive mutations in EPG5 cause Vici syndrome, a multisystem disorder with defective autophagy. Nat Genet 45, 83–7 (2013). 23222957 10.1038/ng.2497 PMC4012842

[b24] Jiang T. *et al.* Nrf2 suppresses lupus nephritis through inhibition of oxidative injury and the NF-kappaB-mediated inflammatory response. Kidney Int 85, 333–43 (2014). 24025640 10.1038/ki.2013.343 PMC3992978

[b25] Zhu L. *et al.* Regulation of Cigarette Smoke (CS)-Induced Autophagy by Nrf2. PLoS One 8, e55695 (2013). 23585825 10.1371/journal.pone.0055695 PMC3621864

[b26] Cho H. Y., Reddy S. P., Yamamoto M. & Kleeberger S.R. The transcription factor NRF2 protects against pulmonary fibrosis. FASEB J 18, 1258–60 (2004). 15208274 10.1096/fj.03-1127fje

[b27] Kobayashi A. *et al.* Oxidative stress sensor Keap1 functions as an adaptor for Cul3-based E3 ligase to regulate proteasomal degradation of Nrf2. Mol Cell Biol 24, 7130–9 (2004). 15282312 10.1128/MCB.24.16.7130-7139.2004 PMC479737

[b28] Zhang D. D., Lo S. C., Cross J. V., Templeton D. J. & Hannink M. Keap1 is a redox-regulated substrate adaptor protein for a Cul3-dependent ubiquitin ligase complex. Mol Cell Biol 24, 10941–53 (2004). 15572695 10.1128/MCB.24.24.10941-10953.2004 PMC533977

[b29] Zhang D. D. & Hannink M. Distinct cysteine residues in Keap1 are required for Keap1-dependent ubiquitination of Nrf2 and for stabilization of Nrf2 by chemopreventive agents and oxidative stress. Mol Cell Biol 23, 8137–51 (2003). 14585973 10.1128/MCB.23.22.8137-8151.2003 PMC262403

[b30] Dinkova-Kostova A. T. *et al.* Direct evidence that sulfhydryl groups of Keap1 are the sensors regulating induction of phase 2 enzymes that protect against carcinogens and oxidants. Proc Natl Acad Sci USA 99, 11908–13 (2002). 12193649 10.1073/pnas.172398899 PMC129367

[b31] Wang X. J. *et al.* Activation of Nrf2 by arsenite and monomethylarsonous acid is independent of Keap1-C151: enhanced Keap1-Cul3 interaction. Toxicol Appl Pharmacol 230, 383–9 (2008). 18417180 10.1016/j.taap.2008.03.003 PMC2610481

[b32] Cai X. *et al.* Frequent mutations in EGFR, KRAS and TP53 genes in human lung cancer tumors detected by ion torrent DNA sequencing. PLoS One 9, e95228 (2014). 24760004 10.1371/journal.pone.0095228 PMC3997391

[b33] Gjyshi O. *et al.* Kaposi’s Sarcoma-Associated Herpesvirus Induces Nrf2 Activation in Latently Infected Endothelial Cells through SQSTM1 Phosphorylation and Interaction with Polyubiquitinated Keap1. J Virol 89, 2268–86 (2015). 25505069 10.1128/JVI.02742-14 PMC4338888

[b34] Ishii T. & Mann G. E. Redox status in mammalian cells and stem cells during culture *in vitro*: critical roles of Nrf2 and cystine transporter activity in the maintenance of redox balance. Redox Biol 2, 786–94 (2014). 25009780 10.1016/j.redox.2014.04.008 PMC4085355

[b35] Jo C. *et al.* Nrf2 reduces levels of phosphorylated tau protein by inducing autophagy adaptor protein NDP52. Nat Commun 5, 3496 (2014). 24667209 10.1038/ncomms4496 PMC3990284

[b36] Moreira P. R. *et al.* Protective effect of bixin on carbon tetrachloride-induced hepatotoxicity in rats. Biol Res 47, 49 (2014). 25299839 10.1186/0717-6287-47-49 PMC4192761

[b37] Fulda S. & Kogel D. Cell death by autophagy: emerging molecular mechanisms and implications for cancer therapy. Oncogene (2015). 10.1038/onc.2014.458. 25619832

[b38] Stohs S. J. Safety and efficacy of Bixa orellana (achiote, annatto) leaf extracts. Phytother Res 28, 956–60 (2014). 24357022 10.1002/ptr.5088

[b39] Auttachoat W., Germolec D. R., Smith M. J., White K. L. Jr. & Guo T. L. Contact sensitizing potential of annatto extract and its two primary color components, cis-bixin and norbixin, in female BALB/c mice. Food Chem Toxicol 49, 2638–44 (2011). 21777644 10.1016/j.fct.2011.07.009

[b40] Tao S. *et al.* Tanshinone I activates the Nrf2-dependent antioxidant response and protects against As(III)-induced lung inflammation *in vitro* and *in vivo*. Antioxid Redox Signal 19, 1647–61 (2013). 23394605 10.1089/ars.2012.5117 PMC3809600

[b41] Wondrak G. T. *et al.* The cinnamon-derived dietary factor cinnamic aldehyde activates the Nrf2-dependent antioxidant response in human epithelial colon cells. Molecules 15, 3338–55 (2010). 20657484 10.3390/molecules15053338 PMC3101712

[b42] Lau A. *et al.* A noncanonical mechanism of Nrf2 activation by autophagy deficiency: direct interaction between Keap1 and p62. Mol Cell Biol 30, 3275–85 (2010). 20421418 10.1128/MCB.00248-10 PMC2897585

[b43] Wang C. H. *et al.* A review of the epidemiologic literature on the role of environmental arsenic exposure and cardiovascular diseases. Toxicol Appl Pharmacol 222, 315–26 (2007). 17433393 10.1016/j.taap.2006.12.022

[b44] Tong K. I. *et al.* Different electrostatic potentials define ETGE and DLG motifs as hinge and latch in oxidative stress response. Mol Cell Biol 27, 7511–21 (2007). 17785452 10.1128/MCB.00753-07 PMC2169061

[b45] Reddy N. M., Potteti H. R., Mariani T. J., Biswal S. & Reddy S. P. Conditional deletion of Nrf2 in airway epithelium exacerbates acute lung injury and impairs the resolution of inflammation. Am J Respir Cell Mol Biol 45, 1161–8 (2011). 21659655 10.1165/rcmb.2011-0144OC PMC3262666

[b46] Cho H. Y. & Kleeberger S. R. Nrf2 protects against airway disorders. Toxicol Appl Pharmacol 244, 43–56 (2010). 19646463 10.1016/j.taap.2009.07.024

[b47] Francis R. C., Vaporidi K., Bloch K. D., Ichinose F. & Zapol W. M. Protective and Detrimental Effects of Sodium Sulfide and Hydrogen Sulfide in Murine Ventilator-induced Lung Injury. Anesthesiology 115, 1012–21 (2011). 21912243 10.1097/ALN.0b013e31823306cf PMC3752661

[b48] Mizushima N. Autophagy: process and function. Genes Dev 21, 2861–73 (2007). 18006683 10.1101/gad.1599207

[b49] Darvekar S. R., Elvenes J., Brenne H. B., Johansen T. & Sjottem E. SPBP is a sulforaphane induced transcriptional coactivator of NRF2 regulating expression of the autophagy receptor p62/SQSTM1. PLoS One 9, e85262 (2014). 24416372 10.1371/journal.pone.0085262 PMC3887019

[b50] Joung I., Strominger J.L. & Shin J. Molecular cloning of a phosphotyrosine-independent ligand of the p56lck SH2 domain. Proc Natl Acad Sci USA 93, 5991–5 (1996). 8650207 10.1073/pnas.93.12.5991 PMC39176

[b51] Jo C. *et al.* Sulforaphane induces autophagy through ERK activation in neuronal cells. FEBS Lett 588, 3081–8 (2014). 24952354 10.1016/j.febslet.2014.06.036

[b52] Ro S. H. *et al.* Sestrin2 promotes Unc-51-like kinase 1 mediated phosphorylation of p62/sequestosome-1. FEBS J 281, 3816–27 (2014). 25040165 10.1111/febs.12905 PMC4156532

[b53] Tong K. I. *et al.* Keap1 recruits Neh2 through binding to ETGE and DLG motifs: characterization of the two-site molecular recognition model. Mol Cell Biol 26, 2887–900 (2006). 16581765 10.1128/MCB.26.8.2887-2900.2006 PMC1446969

[b54] Eskelinen E. L. & Saftig P. Autophagy: a lysosomal degradation pathway with a central role in health and disease. Biochim Biophys Acta 1793, 664–73 (2009). 18706940 10.1016/j.bbamcr.2008.07.014

