# Supplementary material for: Bixin protects mice against ventilation-induced lung injury in an NRF2-dependent manner
Source: Sci Rep. 2016 Jan 5;6:18760. doi: 10.1038/srep18760 (PMC4700431; doi:10.1038/srep18760)
Supplement: Supplementary Information [file srep18760-s1.doc]

**Bixin protects mice against ventilation-induced lung injury in an NRF2-dependent manner**

Shasha Tao^1^, Montserrat Rojo de la Vega^1^, Hector Quijada^2^, Georg T. Wondrak^1,3^, Ting Wang^2*^, Joe G. N. Garcia^2*^, Donna D. Zhang^1,3*^


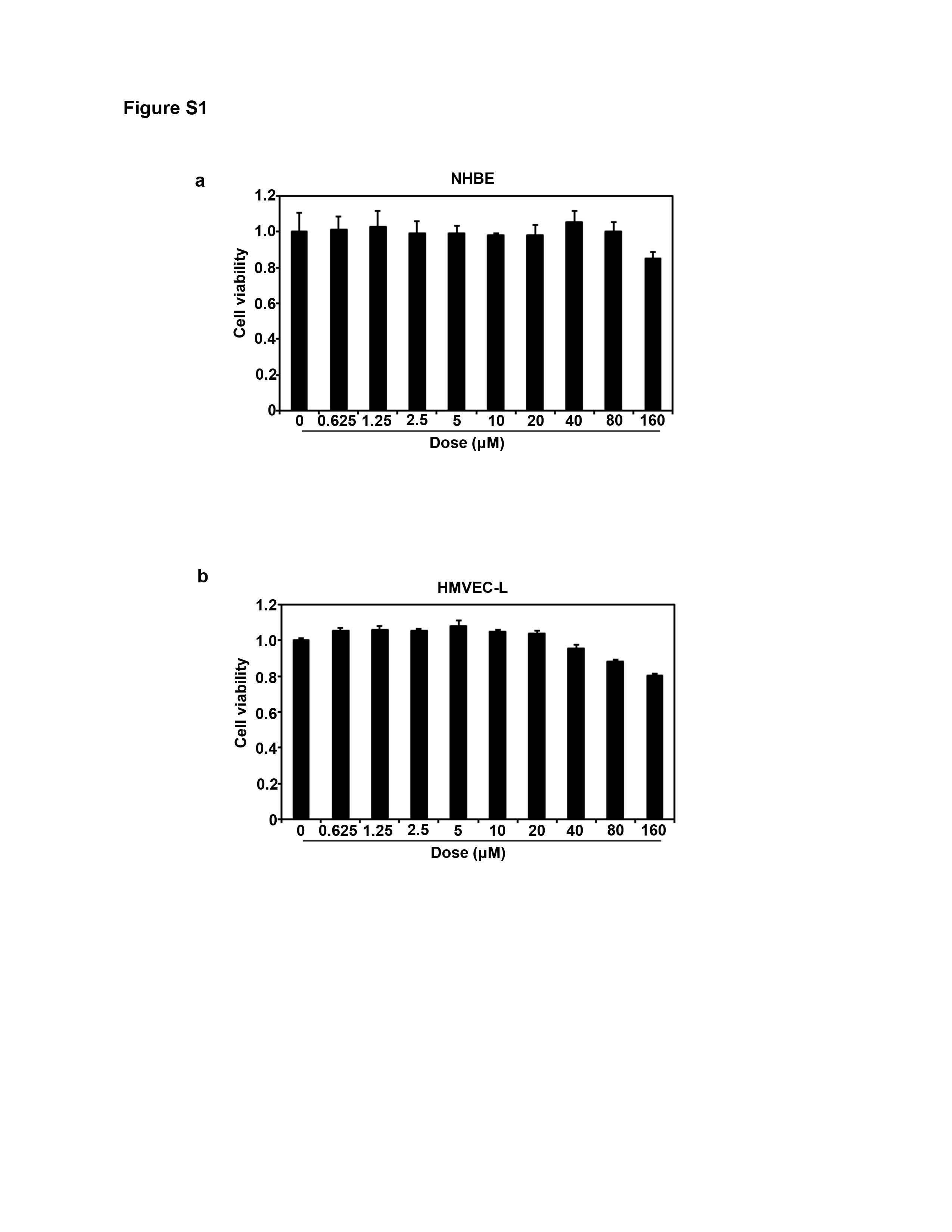


**Figure S1. Bixin cytotoxicity in primary human cell lines.** Cell viability was measured in (a) normal human bronchial epithelial cells (NHBE) and (b) normal lung microvascular endothelial cells (HMVEC-L) treated with the indicated doses of bixin for 48 h.


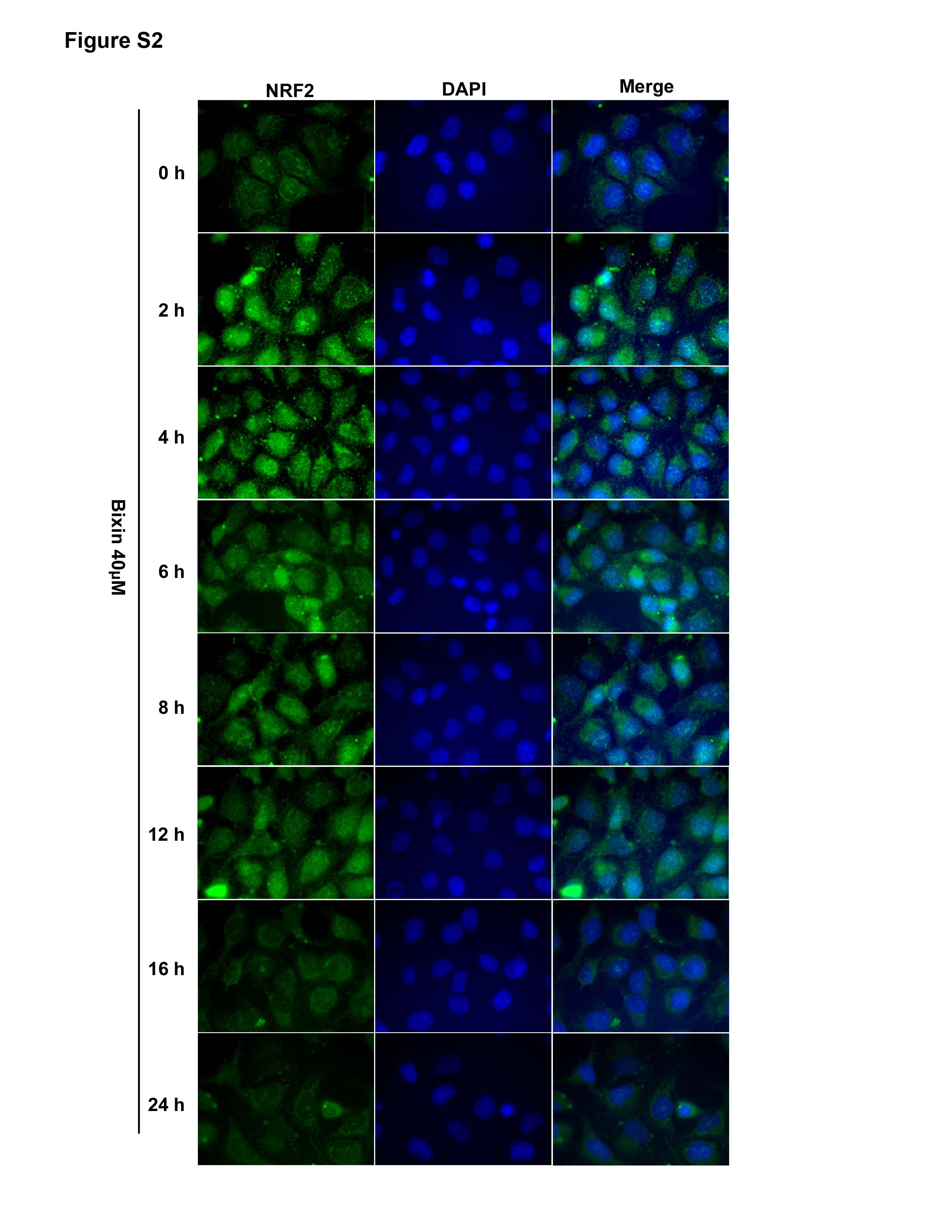


**Figure S2.** H1299 cells were treated with bixin (40 μM) for the indicated time points, fixed and stained. Images were taken using a fluorescence microscope. Representative images are shown.

**
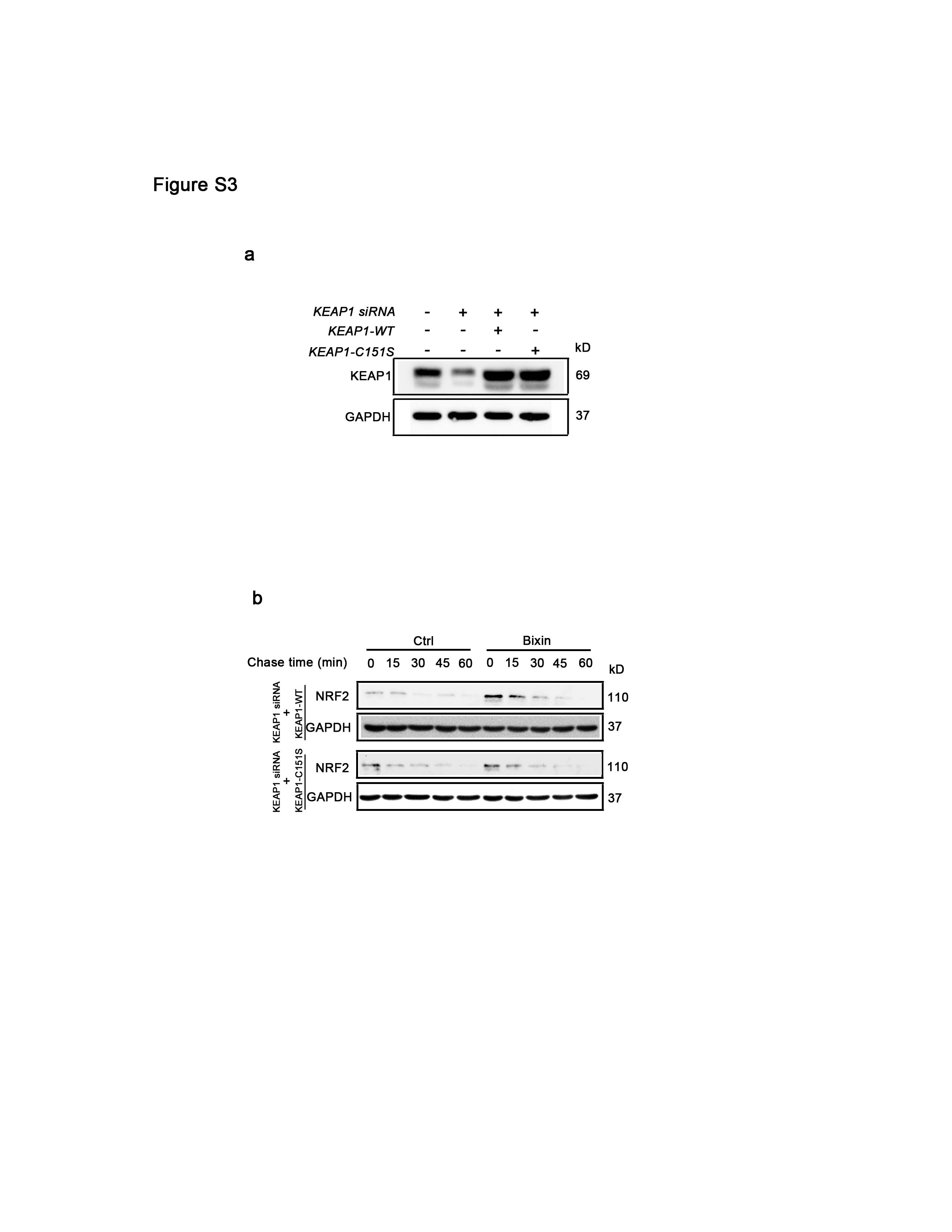
**

**Figure S3.** (a) H1299 cells were transfected with siRNA and 24 h later were transfected with plasmids encoding for KEAP1-WT or KEAP1-C151S for another 24 h. Cell lysates were subjected to immunoblot analyses. (b) H1299 cells were transfected with siRNA and 24 h later were transfected with plasmids encoding for KEAP1-WT or KEAP1-C151S. 24 h later the cells were either left untreated or treated with bixin (40 μM) for 4 h. Cycloheximide (CHX, 50 μM) was added and cells were lysed at the indicated time points. Cell lysates were subjected to immunoblot analysis using NRF2 and GAPDH antibodies.

**
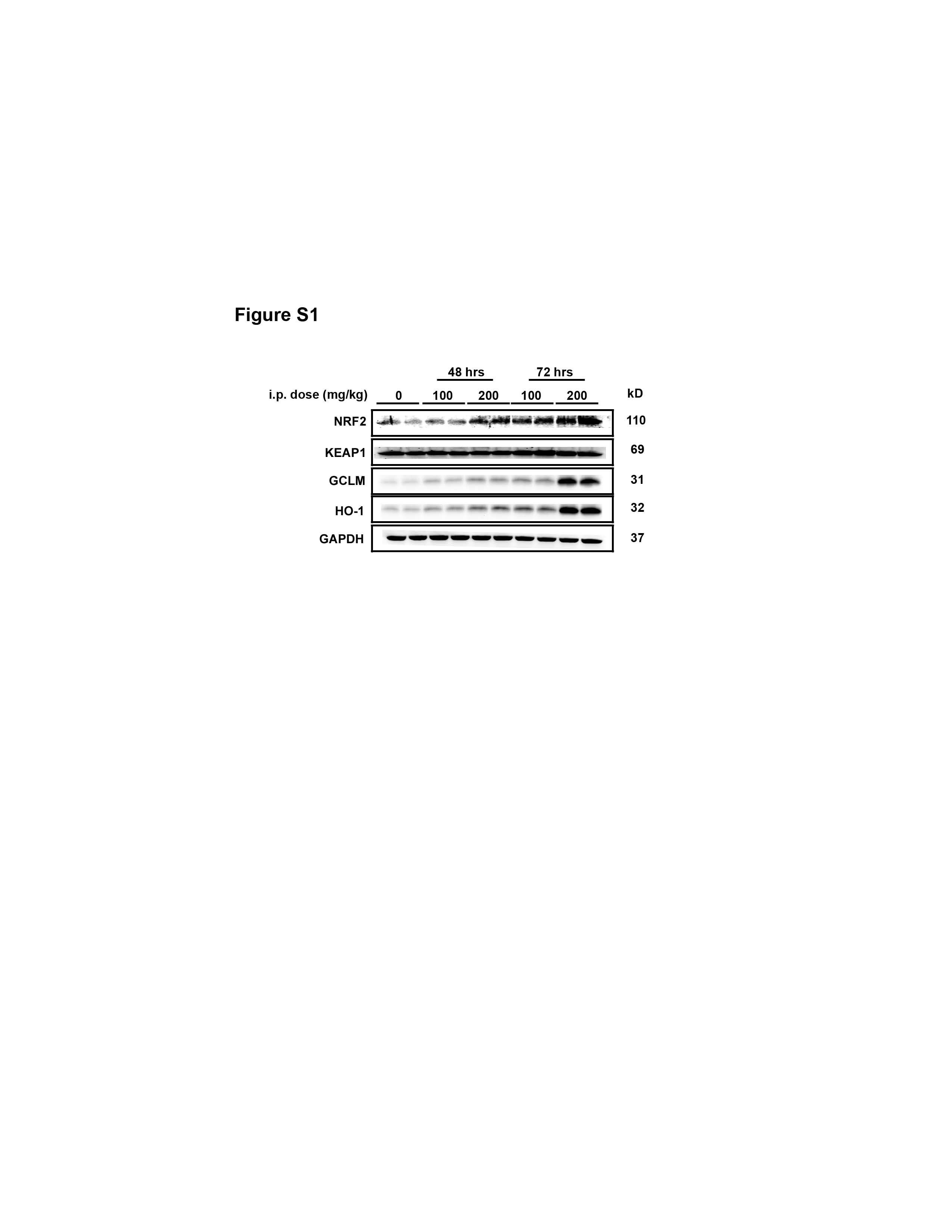
**

**Figure S4. Pulmonary NRF2 signaling pathway activation by IP injection of bixin.** *Nrf2^+/+^* mice were exposed to two doses of bixin (100 and 200 mg/kg) for the indicated time points. Lung tissue lysates were subjected to immunoblot analysis with the indicated antibodies. Each lane represents an individual mouse.
